# Illegal waste sites as potential micro foci of Mediterranean Leishmaniasis

**DOI:** 10.1186/1756-3305-7-S1-O19

**Published:** 2014-04-01

**Authors:** V Ivović, K Kalan, S Zupan, VE Buzan

**Affiliations:** 1 Science and Research Centre, University of Primorska, Koper - Capodistria, Slovenia; 2 Faculty of Mathematics, Natural Sciences and Information Technologies University of Primorska, Koper - Capodistria, Slovenia

## 

Apart from being against the law, illegal waste dumping also poses a threat to human health and to the environment. Solid and decomposing waste is an ideal breeding ground for a number of rodents, insects, and other vermin that pose a health risk through the spread of infectious diseases. The main objective of this study was to survey disease vectors and rodents for the presence of *Leishmania *sp. from illegal waste sites along the Istrian Peninsula in Slovenia and Croatia.

The entomological and rodent survey was carried out between April 2011 and May 2013, at 12 locations, considering only illegal waste sites which consist of at least 2 m^3 ^of garbage.

A total of 119 specimens of Phlebotomine sandflies were collected. Five species were identified as follow: *Sergentomyia minuta *(48.7%), *Phlebotomus perniciosus *(30.3%), *P. papatasi *(13.4%), *P. neglectus *(5%) and *P. mascitii *(2.6%). Additionally, 173 small rodents were trapped at the same sites including following species: *Rattus rattus *(3.5%), *Mus musculus *(44%), *Apodemus agrarius *(27%), *A. flavicollis *(15%) and *A. sylvaticus *(10.5%). A geospatial analysis software ArcView was used to map the distribution of both vectors and rodents.

Sandflies and rodents were screened using a molecular probe to amplify an approximately 120 bp fragment of the kinetoplast DNA (kDNA) minicircle for the detection of *Leishmania *spp. parasites. While not recorded in the tested sandflies, *L. infantum *DNA was detected in the spleen of one juvenile black rat (*R. rattus*).

Despite few published records on *Leishmania *spp. infection in black rats, the addition of our record highlights the importance for further investigation into the frequency and distribution of such occurrences so that we may better classify the role of rodents as potential reservoirs of leishmaniasis in the Mediterranean basin.

